# Supplementary material for: Neem Leaf Extract Exhibits Anti-Aging and Antioxidant Effects from Yeast to Human Cells
Source: Nutrients. 2024 May 16;16(10):1506. doi: 10.3390/nu16101506 (PMC11124485; doi:10.3390/nu16101506)
Supplement: Supplementary file 1 [file nutrients-16-01506-s001.zip › nutrients-2992343-supplementary.pdf]

Table S1. The potential active compounds of neem leaf extract. ("N/A" means "Not applicable").

No.	Compound	Molecular Formula	Adduct	Reference m/z	m/z	Retention time (s)	Superclass	MQScore	Number of targets (yeast)	Number of targets (human)	InChIKey
1	3-{6-[((6S,2R,3R,4R,5R)-3,4,5-trihydroxy-6-methyl(2H-3,4,5,6-tetrahydropyran-2 -yloxy))methyl](2S,4S,5S,3R,6R)-3,4,5-trihydroxy(2H-3,4,5,6-tetrahydropyran-2-yloxy)}-2-(2,3-dihydroxyphenyl)-5,7-dihydroxychromen-4-one	C27H30O16	M-H	609.146	609.139	239.99	Flavonoids	0.977483	0	9	ZMDOPLQSOGZPJN-UHFFFAOYSA-N
2	Nicotiflorin	C27H30O15	M-H	593.151	593.144	251.551	Flavonoids	0.97537	0	12	RTATXGUCZHCSNG-QHWHWDPRSA-N
3	Quercetin	C15H10O7	M-H	301.035	301.031	324.485	Flavonoids	0.967868	213	328	REFJWTPEDVJJIY-UHFFFAOYSA-N
			2M-H	603.078	603.072	327.858		0.952956			
			M+H	303.05	303.051	240.403		0.922939			
4	Sophoraflavanone B	C20H20O5	M-H	339.124	339.119	456.999	Flavonoids	0.958042	10	5	LPEPZZAVFJPLNZ-UHFFFAOYSA-N
5	Quercetin 3-O-malonylglucoside	C24H22O15	M-H	549.089	549.084	256.759	Flavonoids	0.955749	0	11	NBQPHANHNTWDML-UJKBSQBPSA-N
			M+H	551.103	551.105	256.966		0.937842			
6	(+)-Epigallocatechin	C15H14O7	M-H	305.067	305.063	139.791	Flavonoids	0.947082	0		XMOCLSLCDHWDHP-WFASDCNBSA-N

7	Tiliroside	C30H26O13	M-H	593.13	593.124	313.544	Flavonoids	0.942753	4	6	DVGGLGXQSFURLP-VWMSDXGPSA-N
8	N-Acetyl-DL-tryptophan	C13H14N2O3	M-H	245.093	245.09	257.818	Tryptophan alkaloids	0.940713	1		DZTHIGRZJZPRDV-UHFFFAOYSA-N
9	Phloretin	C15H14O5	M-H	273.077	273.074	281.908	Flavonoids	0.940113	54	25	VGEREEWJJVICBM-UHFFFAOYSA-N
10	Isorhamnetin 3-galactoside	C22H22O12	M-H	477.104	477.099	268.077	Flavonoids	0.931247	0	12	CQLRUIIRRZYHHS-UHFFFAOYSA-N
11	Myricetin 3-galactoside	C21H20O13	M-H	479.083	479.078	219.71	Flavonoids	0.927972	0		FOHXFLPXBUAOJM-MGMURXEASA-N
12	5,7-Dihydroxy-2-(4-hydroxy-3-methoxyphenyl)-3-[[3,4,5-trihydroxy-6-(3,4,5-trihydroxy-6-methyloxan-2-yl)oxyoxan-2-yl]methoxy]chromen-4-one	C28H32O16	M-H	623.162	623.156	258.192	Flavonoids	0.925616	0	5	LVAKTTFHRFASRZ-UHFFFAOYSA-N
13	CHEBI:181292	C22H28O3	M-H	339.197	339.196	626.607	Steroids	0.923846	0	4	UJVLDDZCTMKXJK-AFDHUMPMSA-N
14	Decylbenzene sulfonate	C16H26O3S	M-H	297.141	297.149	514.903	Aromatic polyketides	0.915461	0		UAZLASMTBCLJKO-UHFFFAOYSA-N
15	Phlorizine	C21H24O10	M-H	435.13	435.125	260.992	Flavonoids	0.902518	0	4	IOUVKUPGCMBWBT-UHFFFAOYSA-N
16	Licoflavanone	C20H20O5	M-H	339.123	339.12	481.887	Isoflavonoids | Flavonoids	0.902477	0	5	CGKWSLSAYABZTL-UHFFFAOYSA-N
			M+H	341.138	341.14	481.701		0.767127			
17	L-Epicatechin	C15H14O6	M-H	289.072	289.068	194.004	Flavonoids	0.900784	26	10	PFTAWBLQPZVEMU-UHFFFAOYSA-N

18	Trehalose	C12H22O11	M-H	341.109	341.105	38.082	Saccharides	0.895209	43	95	HDTRYLNUVZCQOY-LIZSDCNHSA-N
19	Quercitrin	C21H20O11	M-H	447.093	447.088	260.205	Flavonoids	0.879555	14	35	OXGUCUVFOIWWQJ-HQBVPOQASA-N
20	4-Caffeoylquinic acid	C16H18O9	M-H	353.088	353.084	155.412	Phenylpropanoids (C6-C3)	0.877415	3	2	GYFFKZTYYAFCTR-JUHZACGLSA-N
21	Avicularin	C20H18O11	M-H	433.078	433.074	257.208	Flavonoids	0.867424	0	10	BDCDNTVZSILEOY-UXYNSRGZSA-N
22	Afzelin	C21H20O10	M-H	431.098	431.094	286.49	Flavonoids	0.864259	7	29	SOSLMHZOJATCCP-AEIZVZFYSA-N
23	Isoastilbin	C21H22O11	M-H	449.109	449.104	256.493	Flavonoids	0.858162	0		ZROGCCBNZBKLEL-UHFFFAOYSA-N
24	Kaempferol	C15H10O6	M-H	285.04	285.037	365.345	Flavonoids	0.836085	55	158	IYRMWMYZSQPJKC-UHFFFAOYSA-N
			M+H	287.055	287.056	254.567		0.943702			
25	3-Glu-7-Rha Quercetin	C27H30O16	M-H	609.116	609.118	297.618	Flavonoids	0.822161	1	6	OTUCXMIQUNROBJ-DWZPKPSVSA-N
26	3-p-Coumaroylquinic acid	C16H18O8	M-H	337.093	337.089	185.305	Phenylpropanoids (C6-C3)	0.807546	0	2	BMRSEYFENKXDIS-QHAYPTCMSA-N
27	16-Hydroxy-9-oxooctadeca-10,12,14-trienoic acid	C18H28O4	M-H	307.19	307.187	420.602	Fatty Acids and Conjugates	0.792897	0		KLFMLBSZQZVKDC-UHFFFAOYSA-N
28	MEGxp0_001915	C17H14O7	M-H	329.067	329.063	386.669	Flavonoids	0.761361	0		LOVMOOUQHWMIHZ-RZNTYIFUSA-N
29	6,8-Di-C-glucopyranosylnaringenin	C27H32O15	M-H	595.167	595.161	197.949	Flavonoids	0.755636	0		KLDARLGFVGIIIL-UHFFFAOYSA-N
30	2,3,4-Trihydroxy-5-[3-(4-hydroxyphenyl)prop-2-enoyloxy]hexanedioic	C15H16O10	M-H	355.066	355.063	141.387	Phenylpropanoids (C6-C3)	0.744382	0		LWEUFDJHDOPWFE-UHFFFAOYSA-N

	acid										
31	Xanthohumol	C21H22O5	M-H	353.139	353.135	422.856	Flavonoids	0.71692	13	19	ORXQGKIUCDPEAJ-YRNVUSSQSA-N
32	Dodecylbenzenesulfonic acid	C18H30O3S	M-H	325.173	325.18	587.275	N/A	0.706087	2	1	WBIQQQGBSDOWNP-UHFFFAOYSA-N
33	Procyanidin trimer T2	C45H38O18	M-H	865.199	865.19	190.685	Flavonoids	0.703292	0		MOJZMWJRUKIQGL-UHFFFAOYSA-N
34	N/A	C49H56N4O8	M+2H	415.212	415.213	490.208	N/A	0.98754			OZMHKRBQDGOIDU-UHFFFAOYSA-N
35	Tetradecyldiethanolamine	C18H39NO2	M+H	302.305	302.307	573.779	Fatty acyls∣Fatty amides	0.979801			CPHJEACXPATRSU-UHFFFAOYSA-N
36	Abrine	C12H14N2O2	M+H-CH3NH2	188.074	188.071	163.517	N/A	0.977637		4	CZCIKBSVHDNIDH-NSHDSACASA-N
37	Nonaethylene glycol	C18H38O10	M+H	415.254	415.255	221.113	Glycerolipids	0.976824	44	76	YZUUTMGDONTGTN-UHFFFAOYSA-N
38	Kaempferol-7-O-neohesperidoside	C27H30O15	M+H	595.166	595.168	254.211	Flavonoids	0.964195		1	ZEJXENDZTYVXDP-UHFFFAOYSA-N
39	Kaempferin	C21H20O10	M+H	433.113	433.115	287.412	Flavonoids	0.964082		10	SOSLMHZOJATCCP-UHFFFAOYSA-N
40	Undecaethylene Glycol	C22H46O12	M+H	503.306	503.308	237.063	Glycerolipids	0.960846			PSVXZQVXSXSQRO-UHFFFAOYSA-N
41	N-Lauryldiethanolamine	C16H35NO2	M+H	274.274	274.275	469.958	Fatty acyls∣Fatty amides	0.959429		1	NKFNBVMJTSYZDV-UHFFFAOYSA-N
42	Spiraeoside	C21H20O12	M+H	465.103	465.104	240.562	Flavonoids	0.959397			OIUBYZLTFSLSBY-UHFFFAOYSA-N
43	8-Prenylnaringenin	C20H20O5	M+H	341.138	341.14	457.118	Flavonoids	0.957149	13	9	LPEPZZAVFJPLNZ-SFHVURJKSA-N
44	Vitamin P	C27H30O16	M+H	611.161	611.161	240.015	Flavonoids	0.955041		12	IKGXIBQEEMLURG-UHFFFAOYSA-N

45	Procyanidin B2	C30H26O12	M+H	579.15	579.152	171.631	N/A	0.94619	11	7	XFZJEEAOWLFHDH-NFJBMHMQSA-N
46	Myricetin	C15H10O8	M+H	319.045	319.046	224.47	Flavonoids	0.943855	10	131	IKMDFBPHZNJCSN-UHFFFAOYSA-N
47	Compound NP-000390	C28H32O16	M+H	625.176	625.179	258.55	Flavonoids	0.939295		8	UIDGLYUNOUKLBM-ACHADDEXSA-N
48	1-Palmitoylphosphatidylcholine	C24H50NO7P	M+H	496.339	496.342	585.515	N/A	0.915245	4	14	ASWBNKHCZGQVJV-HSZRJFAPSA-N
49	Acetyl tributyl citrate	C20H34O8	M+H	403.233	403.236	656.655	Fatty esters	0.908293		2	QZCLKYGREBVARF-UHFFFAOYSA-N
50	CHEBI:143757	C22H30O6	M+ACN+H	432.238	432.24	489.593	Diterpenoids	0.876296			WZYGIALDVOKLLL-UHFFFAOYSA-N
51	Acanthoside B	C28H36O13	M+NH4	598.25	598.253	265.01	Lignans	0.868324	1		WEKCEGQSIIQPAQ-IRBNZIFYSA-N
52	Isofraxidin	C11H10O5	M+H	223.06	223.062	206.995	Coumarins	0.868268		6	HOEVRHHMDJKUMZ-UHFFFAOYSA-N
53	LPC(18:3/0:0)	C26H48NO7P	M+H	518.325	518.328	501.054	N/A	0.86329			WKQNRCYKYCKESD-YVHLTTHBSA-N
54	N-Oleoylethanolamine	C20H39NO2	M+H	326.305	326.307	701.164	Fatty amides	0.862799	10	19	BOWVQLFMWHZBEF-KTKRTIGZSA-N
55	Compound NP-012184	C33H40O21	M+H	773.213	773.216	189.1	Flavonoids	0.85147			KVODFZNXODDECO-LSSGXCBUSA-N
56	Beta-gentiobiose octaacetate	C28H38O19	M+H-H2O	325.107	325.115	74.224	N/A	0.838769			GNTLGGDVHFXGLI-QACPWNKNSA-N
57	3,5-Di-tert-butyl-4-hydroxybenzyl alcohol	C15H24O2	M+H-H2O	219.175	219.176	291.499	N/A	0.838441	1	1	HNURKXXMYARGAY-UHFFFAOYSA-N

58	Scopoletin	C10H8O4	M+H	193.05	193.051	197.377	Coumarins	0.837691	3	16	RODXRVNMMDRFIK-UHFFFAOYSA-N
59	Isorhamnetin	C16H12O7	M+H	317.066	317.067	259.771	Flavonoids	0.836521	10	86	IZQSVPBOUDKVDZ-UHFFFAOYSA-N
60	4-O-Glucopyranosyl cinnamate	C15H18O8	M+NH4	344.134	344.135	43.184	Phenylpropanoids (C6-C3)	0.834431	2		LJFYQZQUAULRDF-XMFQKSOQSA-N
61	Flavone Base + 4O, O-MalonylHex	C24H22O14	M+H	535.109	535.11	275.896	Flavonoids	0.831401		2	RNDGJCZQVKFBPI-UHFFFAOYSA-N
62	Pheophorbide A	C35H36N4O5	M+H	593.269	593.277	733.199	Tryptophan alkaloids	0.827008			OINDWIFDMFYGDX-UHFFFAOYSA-N
63	Tryptophan N-glucoside	C17H22N2O7	M+H	367.15	367.152	154.018	N/A	0.824658			ZHBHZDMTVVJASV-JOSVURMMSA-N
64	Syringic acid	C9H10O5	M+H	199.06	199.062	150.957	Phenolic acids (C6-C1)	0.822475	2	12	JMSVCTWVEWCHDZ-UHFFFAOYSA-N
65	Sinapic acid	C11H12O5	M+H-H2O	207.065	207.066	195.241	Phenylpropanoids (C6-C3)	0.818996		8	PCMORTLOPMLEFB-UHFFFAOYSA-N
66	13S-Hydroxy-9Z,11E,15Z-octadecatrienoic acid	C18H30O3	M+H-H2O	277.216	277.217	517.327	N/A	0.815788		2	KLLGGGQNRTVBSU-FQSPHKRJSA-N
67	Dihydroactinolide	C11H16O2	M+H	181.12	181.123	418.062	N/A	0.813538			IMKHDCBNRDRUEB-UHFFFAOYSA-N
68	Compound NP-002265	C27H30O17	M+H	627.155	627.158	189.047	Flavonoids	0.805538		9	UTECWQIXBMWRRR-UHFFFAOYSA-N
69	D-1-[(3-Carboxypropyl)amino]-1-deoxyfructose	C10H19NO7	M+H	266.12	266.125	38.803	N/A	0.80273			HUEOABWGBTXQNF-SFKDOBOXSA-N
70	Flavonol base + 6O, O-MalonylHex	C24H22O16	M+H	567.1	567.102	237.223	Flavonoids	0.791564		9	HPUSNSBFKNJRQA-UHFFFAOYSA-N
71	Pyropheophorbide A	C33H34N4O3	M+H	535.27	535.273	771.657	Tryptophan alkaloids	0.772783		1	IEGUQQKIFBYXLG-UWJYYQICSA-N
72	1-Palmitoyl-Sn-Glycerol	C19H38O4	M+H	331.284	331.287	716.683	N/A	0.771971		1	QHZLMUACJMDIAE-SFHVURJKSA-N

73	Pheophorbide a	C35H36N4O5	M+H	593.274	593.279	731.085	Tryptophan alkaloids	0.771941			NSFSLUUZQIAOOX-LDCXZXNSSA-N
74	Glyceryl monooleate	C21H40O4	M+H	357.3	357.302	726.602	Glycerolipids	0.76615	2	6	RZRNAYUHWVFMIP-UHFFFAOYSA-N
75	(+)-Magnoflorine Iodide	C20H24NO4+	M+	342.17	342.172	227.603	N/A	0.759681		5	YLRXAIKMLINXQY-UHFFFAOYSA-O
76	MEGxp0_001151	C30H36O9	M+H	541.243	541.245	536.342	Triterpenoids	0.758419			NHOIBRJOQAYBJT-WILUFPLZSA-N
77	10-Hydroxyphaeophorbide	C35H36N4O6	M+H	609.272	609.272	704.304	Tryptophan alkaloids	0.750716			TXOKSKCKTYISQV-FHCXDJKBSA-N
78	N-(1-Deoxy-1-fructosyl)phenylalanine	C15H21NO7	M+H	328.14	328.141	117.629	N/A	0.739045			FAVRCIXPIVJIPN-VJDSNFAGSA-N
79	Bolusanthol C	C25H28O5	M+H	409.201	409.203	561.03	Isoflavonoids	0.730126		1	CEBSROOTTDEPKN-UHFFFAOYSA-N
80	Erucamide	C22H43NO	M+H	338.341	338.344	823.353	N/A	0.722795	2	1	UAUDZVJPLUQNMU-KTKRTIGZSA-N
